# Small Pox

**Published:** 1882-02-20

**Authors:** 


### *SMALL-POX.*

The unusual prevalence of small-pox in the United States during the last two or three months, especially in the Northern and Northwestern sections of the Union, has been made sufficiently known through the newspaper press of the country. The disease which at first was alarmingly threatening seems now to be somewhat under control, and if the work of vaccination goes on as it should, we may expect that the disease will soon disappear altogether. We have been questioned in relation to the choice between the bovine and the *humanized* virus, etc.

The *bovine* from healthy young heifers is now generally preferred. Yet many regard the humanized virus, one or two removes from the cow, through healthy young subject, as preferable, and less apt to give rise to erysipelatious or excessive inflammation.

The *bovine* virus as cultivated in the cow and obtained from the vaccine farms—several of which exist in the Northern sections of the Union—is not procured, as many suppose, from inoculating the cow with the virus of small-pox, but is propagated by the insertion of cow-pox lymph from cow to cow.

Nor is it now believed that the original cow-pox, as first described by Jenner, is the result of small-pox or varioa in the cow, but is a variety of the same species as variola.

If it is small-pox in the cow, it is certainly modified in some mysterious way by the unknown method of its communication to the animal, as efforts to produce it by inoculation have not been successful. While a few experimenters have claimed that such was the case, the large proportion of those who have tried it have found that the virus obtained by inoculating the cow with small-pox lymph is not the same as that obtained from the natural vaccina or cow-pox, but is unsafe and liable to produce small-pox instead of vaccina.

Upon the whole, then, we regard the natural cow-pox lymph, or the humanized virus not too far removed, as the best.

